# Editorial: Practical recommendations and consensus for the management of immune mediated hematologic diseases

**DOI:** 10.3389/fimmu.2024.1364227

**Published:** 2024-01-29

**Authors:** Bruno Fattizzo, Sigbjorn Berentsen, Wilma Barcellini

**Affiliations:** ^1^ Fondazione Istituto di Ricovero e Cura a Carattere Scientifico (IRCCS) Ca’ Granda Ospedale Maggiore Policlinico, Milan, Italy; ^2^ Department of Oncology and Hemato-Oncology, University of Milan, Milan, Italy; ^3^ Department of Research and Innovation, Haugesund Hospital, Haugesund, Norway

**Keywords:** paroxysmal nocturnal hemoglobinuria, warm autoimmune hemolytic anemia, cold agglutinin disease, immune thrombocytopenia, target therapy

In the last decade, growing attention has been paid to the so-called “classic” hematologic conditions, including immune mediated diseases. The latter encompass autoimmune cytopenias, namely autoimmune hemolytic anemia (AIHA) and immune thrombocytopenia (ITP), autoimmune neutropenia as well as bone marrow failures including paroxysmal nocturnal hemoglobinuria (PNH). A deeper understanding of the pathogenic mechanisms led to the development of several novel target therapies (1–3). The latter represent an opportunity but also a challenge for the treating physician, given the scarcity of practical recommendations and guidelines.

In this Research Topic we collected five articles spanning in the field of classic hematology: from a “difficult” PNH case successfully treated with a second-generation proximal complement inhibitor (Fattizzo et al.), to broader reviews scanning the horizon of new treatments for AIHA (Berentsen et al.) and ITP (Xiao and Murakhovskaya). Main pathogenic targets and novel treatments are summarized in **Figure 1**. Additionally, Mulder et al. focused on the huge unmet need of managing AIHA in the very acute setting, and Baldari et al. reported on thrombotic complications following splenectomy in autoimmune cytopenias.

**Figure 1:**
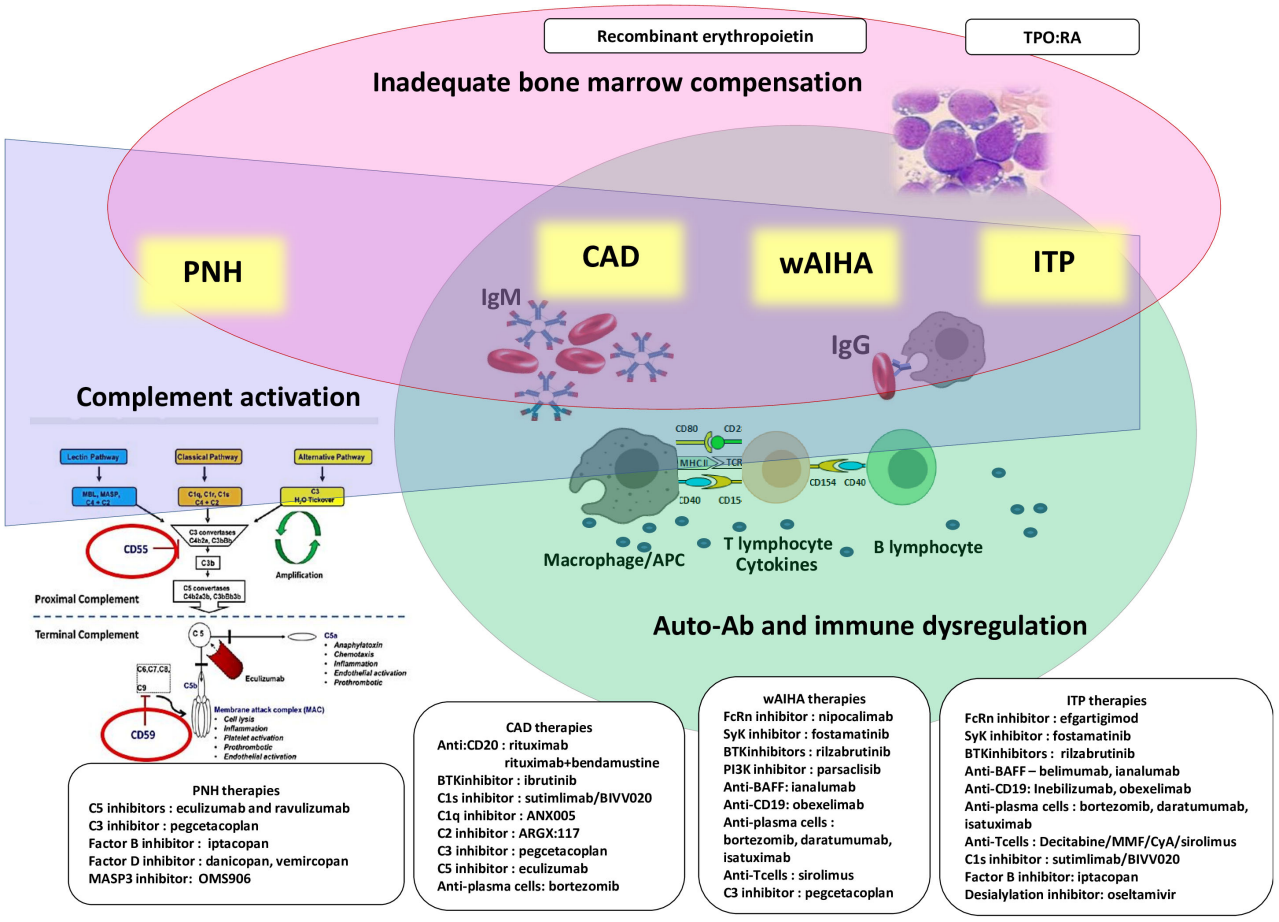
Pathogenic mechanisms and therapeutic targets of immune mediated cytopenias and paroxysmal nocturnal hemoglobinuria (PNH). All diseases display some degree of impaired bone marrow compensation (pink) and complement activation (blue). Complement activation is maximal in PNH and cold agglutinin diseases, CAD, and lower in warm autoimmune hemolytic anemia, wAIHA, and immune thrombocytopenia, ITP). Autoantibodies (Ab) and immune-dysregulation are typical of CAD, wAIHA and ITP (green).

PNH is characterized by the acquisition of a PIG-A gene mutation at the stem cell level, leading to loss of glycosyl- phosphatidyl- inositol anchored complement regulators and cell vulnerability to complement mediated damage with intravascular hemolytic anemia (IVH) (1). For ages, PNH has been treated with transfusions for severe hemolysis and anticoagulants to counteract thrombotic risk, the major cause of mortality. Since late 2000s, the implementation of C5 inhibitor eculizumab revolutionized treatment by efficiently treating anemia, abating thrombotic risk and improving survival. The acknowledgement that more than 2/3 of patients remain anemic despite eculizumab, and that some of them require transfusions and experience breakthrough hemolytic events (BTH) upon complement amplifying conditions boosted the investigation of novel mechanisms of disease. Risitano et al.. highlighted that C5 inhibition led to iatrogenic accumulation of C3 fractions on erythrocyte surface thus leading to C3 mediated extravascular hemolysis in the liver (EVH). Proximal complement inhibitors, recently reviewed elsewhere (1), targeting C3 or the alternative pathway upstream, were developed showing impressive control on EVH in PNH suboptimal responders to C5 inhibitors, as well as of IVH in naïve subjects. The case report included in this Research Topic (Fattizzo et al.) describes the successful use of the C3 inhibitor pegcetacoplan in a PNH patient suffering transfusion dependent anemia despite eculizumab. This drug, FDA and EMA approved, does not however solve the issue of BTH which may be catastrophic due to the sparing of a larger PNH clone (4) and its management is still an unmet need. In fact, the increased use of proximal inhibitors is highlighting a novel category of “suboptimal responders” that we will have to recognize and manage in the next future.

In AIHA, the increased erythrocyte turnover is mediated by autoimmune mechanisms including IgG mediated and complement driven EVH in “warm” forms (wAIHA) and cold agglutinin disease (CAD), respectively. As reviewed in this Research Topic (Berentsen et al.), corticosteroids are the first-line therapy in wAIHA, whilst CAD requires treatments targeting the B-cell underlying lymphoproliferation or the classical complement pathway. The C1s inhibitor sutimlimab represents the first FDA/EMA approved drug for CAD improving hemolysis and anemia in >80% of patients, but not abolishing cold agglutinin production and cold-induced peripheral symptoms, and requiring long-term administration (5). These caveats will require further investigation to determine the possible combination of sutimlimab with B-cell targeting agents and the use “on demand” during disease exacerbations (i.e. colder seasons, complement amplifying conditions, etc.). Furthermore, novel investigational approaches to severe wAIHA are discussed, including the upfront addition of rituximab to prednisolone, and newer agents modulating B-cells (small molecules inhibiting BTK and PI3K, anti-BAFF and bispecific monoclonal antibodies), EVH (the SYK inhibitor fostamatinib), or removing IgG from the circulation (i.e. the neonatal Fc receptor inhibitor nipocalimab). Some of these agents, particularly complement inhibitors and neonatal Fc receptor inhibitors, act very quickly and may be particularly useful during acute AIHA exacerbations. This topic is further expanded by (Mulder et al.) Recent series of AIHA patients such acute and severe as to require admission to the intensive care unit, may exhibit a mortality of around 13% in a couple of days due to extreme anemia with end stage organ failure despite transfusions, steroids, rituximab, plasma exchange and immunosuppressors (6). Hyperhemolysis is often accompanied by IVH and complement consumption, both in wAIHA and CAD, thus hinting a possible utility of complement inhibitors. Additionally, more than 90% of these acute patients had an insufficient bone marrow compensation in a recent study (6) which may be effectively counteracted with the use of recombinant erythropoietin (7). A relatively old drug used in anemia of myelodysplastic syndromes, able to improve anemia in >70% of cases as soon as at 15 days from first dosing and without further immunosuppression.

Similarly to wAIHA, ITP pathophysiology encompasses IgG driven platelet destruction with un underlying broader innate and adaptive immune dysregulation, as well as megakaryocyte dysfunction. Steroids are the front-line treatment and thrombopoietin receptor agonists (TPO-RA), rituximab, fostamatinib, and splenectomy are second/further options (3). Such drugs report similar but heterogeneous efficacy across different settings (i.e. young versus old patient, primary versus secondary ITP, newly diagnosed/persistent versus chronic etc.) and risk of complications, particularly thrombosis for TPO-RA and splenectomy. Although we are definitely in the TPO-RA era (efficacy >70% and sustained remission of therapy in about 1/3 of cases), rituximab has the added benefit of inducing treatment-free remission in over 50% of patients after a defined short-term course. However, Xiao and Murakhovskaya discussed that responses are not long-lasting, and resistance is frequent. Novel inhibitors of FcγR pathway (fostamatinib), FcRn (efgartigimod), complement (sutimlimab), B cells (BTKi and anti-BAFF), and plasma cells (i.e. anti-CD38 monoclonal antibodies) might be useful to overcome such resistance. The risk of thrombosis following splenectomy was further addressed by Baldari et al. who reported 22 patients splenectomised from 2017 to 2023, including 6 ITP. Six patients (27%) developed peri-surgical porto-spleno-mesenteric venous thrombosis, all completely resolving with anticoagulation.

Overall, this plethora of targeting agents will likely make it possible to individualize therapy, based on the disease profile and patient characteristics, maximizing efficacy and avoiding toxicity. In-between forms, such as mixed and Coombs negative AIHA, and rarer associations, as Evans syndrome and autoimmune cytopenias secondary to inborn errors of immunity and hematologic neoplasms, represent a further unmet need for diagnosis and management well outside current recommendations and generally excluded from clinical trials.

## Author contributions

BF: Conceptualization, Data curation, Investigation, Methodology, Supervision, Writing – original draft, Writing – review & editing. SB: Conceptualization, Investigation, Writing – original draft, Writing – review & editing. WB: Conceptualization, Investigation, Writing – original draft, Writing – review & editing.
